# Relative deprivation and mortality – a longitudinal study in a Swedish population of 4,7 million, 1990–2006

**DOI:** 10.1186/1471-2458-12-664

**Published:** 2012-08-16

**Authors:** Monica Åberg Yngwe, Naoki Kondo, Sara Hägg, Ichiro Kawachi

**Affiliations:** 1 Centre for Health Equity Studies (CHESS), Stockholm University/Karolinska Institutet, SE-106 91, Stockholm, Sweden; 2 Department of Health Economics and Epidemiology Research, School of Public Health, The University of Tokyo, 7-3-1 Hongo, Bunkyo-ku, Tokyo, 113-0033, Japan; 3 Department of Medical Epidemiology and Biostatistics, Karolinska Institutet, Stockholm, Sweden; 4 Department of Society, Human Development, and Health, Harvard School of Public Health, Boston, MA, USA

**Keywords:** Relative deprivation, Mortality, Income inequality, Yitzhaki index, Sweden

## Abstract

**Background:**

Relative deprivation has previously been discussed as a possible mechanism underlying the income-health relation. The idea is that income matters to the individual’s health, over and above the increased command over resources, as the basis of social comparisons between a person and his or her reference group. The following study aimed to analyze the role of individual-level relative deprivation for all-cause mortality in the Swedish population. The Swedish context, characterized by relatively small income inequalities and promoting values as egalitarianism and equality, together with a large data material provide unique possibilities for analyzing the hypothesized mechanism.

**Methods:**

The data used are prospective longitudinal data from the Swedish population and based on a linkage of registers. Restricting selection to individuals 25–64 years, alive January 1st 1990, gave 4.7 million individuals, for whom a mortality follow-up was done over a 16-year period. The individual level relative deprivation was measured using the Yitzhaki index, calculating the accumulated shortfall between the individual’s income and the income of all other’s in the person’s reference group. All-cause mortality was used as the outcome measure.

**Results:**

Relative deprivation, generated through social comparisons, is one possible mechanism within the income and health relation. The present study analyzed different types of objectively defined reference groups, all based on the idea that people compare themselves to similar others. Results show relative deprivation, when measured by the Yitzhaki index, to be significantly associated with mortality. Also, we found a stronger effect among men than among women. Analyzing the association within different income strata, the effect was shown to be weak among the poorest. Revealing the importance of relative deprivation for premature mortality, over and above the effect of absolute income, these results resemble previous findings.

**Conclusion:**

Relative deprivation, based on social comparisons of income, is significantly associated with premature mortality in Sweden, over and above the effect of absolute income. Also, it was found to be more important among men, but weak among the poorest.

## Background

Income enables people to purchase the goods and services they need to maintain health – e.g. food, clothing, shelter. Consequently, lack of income (or income poverty) is associated with worse health status [1]. Beyond this direct relationship between income and command over resources, researchers have posed the question of whether an individual’s income relative to other people’s income might also influence his/her level of health. Broadly speaking, the relative income hypothesis has been empirically examined in two streams of research. One stream has sought to test the relationship between aggregate income distribution and health [2,3]. Researchers belonging to this school have been interested in answering the question of whether an individual with a given level of income would experience different health outcomes depending on the distribution of incomes of other people living in the same society. A meta-analysis of 28 multi-level studies suggests that there is a “contextual” effect of income inequality on poor health that is independent of an individual’s level of income. On average, a 0.05 unit increase in the Gini coefficient (a standard measure of income inequality) is associated with roughly an 8\% increase in the risk of mortality [3]. However, how these studies should be interpreted continues to be debated, owing to the possibility of residual confounding by the unobserved characteristics of societies that have high or low income dispersion.

A second stream of research on the relative income hypothesis has sought to focus on the individual-level relationships between the concept of “relative deprivation” and health status. The theory of relative deprivation dates back to WG Runciman [4], who argued that having less income relative to some comparison (or reference) group can lead to feelings of frustration and injustice. Researchers taking this approach have sought to demonstrate that, over and above the increased command over resources, income matters to individuals because it is the basis of social comparisons between a person and his/her reference group [5-8]. Social comparisons have previously been analyzed as a possible mechanism underlying the income-health relation [7-9], and have been linked with happiness [10] as well as outcomes such as deviant behaviour among adolescents [11].

Drawing on Runciman’s theory, Yitzhaki [12] proposed an index for estimating relative deprivation, calculating the accumulated shortfall between one’s own income and the incomes of all others in one’s reference group. Recent studies have used the Yitzhaki index when analyzing the importance of individual relative deprivation for health [5,6,13-17]. Studies in the US have generally reported evidence in support of the hypothesis that relative deprivation is associated with increased risks of mortality [15], mental health services utilization [14], and poor self-rated health [13]. In Japan, Kondo and colleagues studied the association between relative deprivation, measured by the Yitzhaki index, and self-rated health [5]. The overall result indicated that relative deprivation was associated with poor self-rated health, regardless of the reference group used and independent of absolute income. By contrast, studies based in the UK have failed to demonstrate an association between relative deprivation and health [16,17].

The goal of the present study was to conduct an empirical test of relative deprivation and mortality in Sweden – a society that is characterized by a strong egalitarian ethos, as well as a relatively flat degree of income dispersion (in contrast to countries such as the US or UK) [18]. According to Bernburg [11], the individual experience of relative deprivation may be more prominent in a context that promotes values such as egalitarianism, upward social mobility, equal opportunity and individual achievement. In the present study, we therefore sought to analyze the importance of individual-level relative deprivation, as measured by the Yitzhaki index, for all-cause mortality in the Swedish population.

## Methods

### Data

We used prospective longitudinal data from the Swedish population with baseline collection in 1990 and mortality follow-up until 2006. The Swedish Work and Mortality Data Base, maintained at the Centre for Health Equity Studies, was created to study how work, income and labour market position combine to impact morbidity and mortality. The data were based on a linkage of the National Population and Housing Censuses, the Total Population Register (RTB), the Longitudinal Data Base on Education, Income and Employment (LOUISE), the Cause of Death Register and inpatient registers. When restricting the data to individuals alive January 1 1990 and aged 25–64 years, the data included a total of 4.7 million individuals, for whom we conducted a mortality follow-up until 2006 [see Additional file 1 for sample size]. From this cohort, we excluded all individuals who lacked income data, all individuals who had emigrated and for whom we do not have any death data (N = 4 227 490). The occupation variable derives from the 1990 National Population and Housing Census, and 19% had either unclassifiable occupations or missing values, for example persons on sick leave, students or unemployed. These 19% were excluded when using occupation in the models. Ethical permission (no 02–481) was provided by the Regional Ethics Committee at Karolinska Institute in Stockholm.

### Relative deprivation

The central exposure variable, the measure of relative deprivation was calculated using the Yitzhaki index [12], in a similar way as done in previous studies [5,13,15]. The index calculates the relative deprivation for each individual by summarizing the differences in income between that individual and all individuals with a higher income in that person’s reference group, taking the size of the reference group into consideration. Relative deprivation for the individual i is the sum of the income gap between i and j (yj – yi, where j has a higher income than i), divided by number of people in the reference group (N). Relative deprivation (i) = 1/N ∑ (yj – yi), ∀ yj > yi.

The income measure used in the Yitzhaki index calculation is the disposable individual income, based on the individual’s total income from work and social benefits minus taxes. To simplify the interpretation of the results, the Yitzhaki index was rescaled by division of 10 000 SEK. When constructing reference groups for each individual, we made the assumption that individuals compare themselves to similar others. In the analysis, we tested different reference group settings in accordance with previous studies [5,13]: 1) Living region (divided into metropolitan areas 36%, large towns 33% and other areas including countryside 31%), 2) Occupation (classified in accordance with the Swedish Socioeconomic Classification (SEI) and divided into the categories higher non-manual employees, lower non-manual employees, qualified manual workers, unqualified manual workers, self-employed and farmers. The final two, self-employed and farmers were categorized together), 3) Age group (25–34, 35–44, 45–54, 55–64) and 4) a combination of these three.

### Covariates

In the analysis, we included age, marital status (married, unmarried, divorced and widowed), occupation (higher non-manuals, lower non-manuals, qualified manual, unqualified manual, self-employed and farmers) and income as covariates.

### Statistical analysis

We used Cox regression modeling (SAS version 9.2) with survival time in years as the time scale, with a maximum of 16 years. All analyses were stratified by gender, as previous studies have shown a gender difference in the importance of income and relative deprivation [7,19]. All people still alive in 2006 were censored. We calculated the crude model using the different reference group settings from our complete data set. Estimates are presented as hazard ratios (HR) with 95% confidence intervals. In the multivariate models, we adjusted for age group and marital status in the first model (data not shown). In Model 2 we adjusted for age group, marital status and occupation, and in Model 3 we also added income.

## Results

The descriptive analyses in Table 1 show the number of deceased by age group, marital status and occupational group. The number of deceased is higher among males than females. The proportion of deceased during the follow-up also varies across marital status and occupation, with higher rates among divorced, widows/widowers and unqualified manual workers. Descriptive analyses also show the median relative deprivation within reference groups used. For an individual, having a lower value on the relative income deprivation index means having a higher income within the reference group. Having a value of 0, means having the highest income within the specific reference group. The median relative income deprivation differs depending on whether living region, age group or occupation has been used. The lowest value was found when combining these three, since the income diversity is smaller when reference groups become more specific.

**Table 1:** Sociodemoqraphic characteristics in Swedish men and women

**Variable**	**Male**				**Female**			
	**No. (%) totals**	**No. (%) deceased**	**P.years**	**Mo. rate**	**No. (%) totals**	**No. (%) deceased**	**P. years**	**Mo. rate**
Age *group (years old)*								
25-34	578 779 (27.2)	12 242 (2.1)	9 358 400	0.0007	552 756 (26.4)	654 (1.21	8 849 328	0,0001
35-44	610 249 (28.6)	30 115 (4.9)	10 004 904	0,0015	594 442 (28.4)	18 965 (3.2)	9 662 792	0,0010
45-54	538 449 (25.3)	62 440 (11.6)	9 114 704	0,0035	522 613 (24.9)	38 724 (74)	8 671 600	0,0023
55-64	404 251 (19 0)	123 231 (30 5)	7 453 864	0, 0083	425 951 *(2 fl* 2)	80 072 (18.8)	7 455 792	0,0054
Marital status								
Marned	1 220 437 (57.3)	124 481 (10.2)	20 522 840	0, 0031	1 301 392 (62.1)	83 466 (6.4)	21 490 0000	0,0020
Unmarried	69 625 (32.7)	57 792 (8.3)	1 576 336	0.0184	477 097 (22.8)	21 714 (4.6)	7 807 264	0,0014
Divorced	197 398 (9.3)	40531(20.5)	3482616	0,0058	245 331 (11.7)	26 176 (10.7)	4 134 704	0,0032
Widow/widower	17 643 (0.8)	5224 (29.6)	324 080	0,0081	71 942 (3.4)	12945 (18.0)	1 254632	0,0052
*Occupation*								·
Higher non-manual	596 898 (34.2)	41146 (6.9)	9 879 536	0,0021	481 123 (29.1)	18 31 2 (3.8)	7 844 464	0,001 2
Lower non-manual	182 953 (10.5)	16 621 (9.1)	3 060 216	0,0027	376 519 (22.7)	19 916 (5.3)	6 183 632	0,0016
Qualified manual	394 719 (22.6)	31194 (7.9)	6 565 040	0,0024	181 864 (11.0)	7 002 (3.9)	2 965 840	0,0012
Unqualified manual	413 776 (23.7)	39 328 (9.5)	6 935 040	0,0029	545 996 (33.0)	3 333 (6.1)	8 762 600	0,0002
Self-employed, farmers	159 665 (9.1)	16 018 (10.0)	2 682 784	0,0030	70 523 (4.3)	3 995 (5.7)	1 160 328	0,0017
	Median[25 %75 %]				Median[25 %75 %]			:
*Disposable income (10,000 SEK)*	11.97 [9.74 14.44]				9.34 [7.37 11.38]			
*Relative income deprivation by* Yitzhaki *index and* by the *definition of reference group (10,000 SEK)*								
Living region	1.163[0.5662250]				2495[1 4442925]			
Age group	1.175 [0.576 2.220]				2.480 [1.399 3.9401			
Occupation	0.999 [0.463 1.897]				2.224 [1.251 3.523]			
Living region, age group								
and occupation	0.949 [0.431 1.843]				2.166 [1.216 3.423]			

P.years- Person years, Mo. rate - Mortaly rate.

In order to illustrate the possibility of non-linearity in the association between relative deprivation and mortality, we calculated HR for continuous as well as quartiled relative deprivation (Table 2). Cox regression showed that, among men, relative deprivation was significantly associated with mortality regardless of the definition of reference groups for which we calculated relative deprivation. The crude HRs for mortality by 10 000 SEK unit increase in relative deprivation are between 1.069 (95% confidence intervals [CI]: 1.066 -1.072) and 1.109 (95%CI: 1.107-1.111), depending on the reference group. The crude HRs for women range between 0.986 (0.982-0.990) and 1.118 (1.116 – 1.120). The crude HR of the highest versus the lowest quartile is between 1.414 (95%CI: 1.392-1.437) and 2.503 (95%CI: 2.474-2.532) among men, and between 0.958 (95%CI: 0.937 - 0.980) and 2.082 (95%CI: 2.041 - 2.123) among women, depending on the reference group used. Adjusting for age group, marital status and occupation, the HR of the top vs. bottom quartile among men is attenuated to the values ranging between 1.693 and 1.821, but still statistically significant. Among women, these results vary between 1.127 (95%CI: 1.097-1.157) and 1.182 (95%CI: 1.151-1.213). In Model 3, we also adjust for absolute income. Although this additional adjustment attenuates the association between relative deprivation and mortality, the HRs remain statistically significant among men (HRs were between 1.668 and 1.751), whereas the HRs among women largely attenuate to lower values (between 0.992 and 1.155).

**Table 2:** Crude and adjusted hazard ratios (95 % confidence intervals) for overall mortality by the level of relative deprivation in Swedish men and women

	**Reference group defined by**							
	**Living region**		**Age group**		**Occupation**		**Living region, age group and occupation**	
*Male*								
Crude								
Continuous	1109 (1107–1111)	p < .0001	1.104 (1.102-1.10 6)	p < .0001	1069 (1.066-1072)	p < .0001	1.090 (1.087-1.093)	p < .0001
Top vs bottom quartile	2503 (2.474-2.532)	p < .0001	2.491 (2.467-2.52 7)	p < .0001	1414 (1 .392-1 437)	p < .0001	1.587 (1 .563-1.612)	p < .0001
Model 2								
Continuous	1.101 (1.097-1.105)	p < 0001	1.103 (1.099-1.10 7)	p < .0001	1.105 (1.101-1 109)	p < .0001	1.107 (1.103-11 11)	p < .0001
Top vs bottom quartile	1.746 (1.711-1.782)	p < .0001	1.821 (1.784-1.85 9)	p < .0001	1.693 (1.663-1 724)	p < .0001	1.749 (1.719-1779)	p < .0001
Model 3								
Continuous	1.079 (1.074-1.085)	p < .0001	1.060 (1.074-1.08 5)	p < .0001	1.090 (1.084-1 095)	p < .0001	1.096 (1.091-1101)	p < .0001
Top vs bottom quartile	1.668 (1.627-1.710)	p < .0001	1.749 (1.703-1.79 5)	p < .0001	1.694 (1.662-1 727)	p < .0001	1.751 (1.719-1.784)	p < .0001
*Female*								
Crude								
Continuous	1118 (1.116-1.120)	p < .0001	1.098 (1.096-1.100)	p < .0001	0.986 (0.982-0.990)	p < .0001	1.002 (0.998-1.006)	p = .35
Top vs bottom quartile	2.082 (2.041 -2.123)	p < .0001	2.051 (2.012-2.092)	p < .0001	0.989 0.966-1 012)	p = .34	0.958 (0.937-0.980)	p = .0002
Model 2								
Continuous	1.031 (1.027-1.036)	p < .0001	1.027 (1.022-1.03 2)	p < .0001	1.024 (1.019-1.028)	p < .0001	1.029 (1.025-1.034)	p < .0001
Top vs bottom quartile	1.182 (1.151-1213)	p < .0001	1.127 (1.097-1.157)	p < .0001	1.133 (1.105-1 162)	p < .0001	1.159 (1.130-1.188)	p < .0001
Model 3								
Continuous	1.027 (1.019-1.034)	p < .0001	1.009 (0.999-1.019)	p = .06	1.003 (0.994-1.013)	p = .51	1.025 (1.017-1.032)	p < .0001
Top vs bottom quartile	1.155 (1.110-1202)	p < .0001	0.992 (0.944-1.042)	p = .74	1.044 (1.000-1 090)	p = .05	1.101 (1.060-1.144)	p < .0001

To further disentangle the relationship between relative deprivation and income, we calculated HRs for mortality by level of relative deprivation within income quartiles (Table 3). In males, this clearly illustrates the differences in HR depending on income level where individuals in the lowest quartile are not affected by relative deprivation. The multicolinearity between relative deprivation and income was tested by calculating the correlation coefficient and revealed a moderate overall association (r = − 0.45 Y index age groups; r = −0.45 Y index living region; r = −0.50 Y index occupation).

**Table 3:** Crude and adjusted hazard ratios (95 % confidence intervals) for overall mortality by the level of relative deprivation in Swedish men and women by income quartiles Q1-Q4

	**Reference group defined by**							
	**Living region**		**Age group**		**Occupation**		**Living region, age group and occupation**	
*Male*								
Continuous Q1	1.002 (0.994-1 .010)	p = .62	0.996 (0.987-1.005)	p = .35	0.992 (0.983-1.001)	p = .08	1.001 (0.992-1.009)	p = .91
Continuous Q2	1.241 (1.219-1.264)	p < .0001	1.363 (1.325-1.402)	p < .0001	1.321 (1.287-1.356)	p < .0001	1.240 (1.217-1.264)	p < .0001
Continuous Q3	1.228 (1.202-1.255)	p < .0001	1.243 (1.199-1.289)	p < .0001	1.195 (1.159-1.233)	p < .0001	1.180 (1.157-1.203)	p < .0001
Continuous Q4	1258 (1.220-1.297)	p < .0001	1.309 (1.260-1.359)	p < .0001	1.169 (1.145-1.194)	p < .0001	1.133 (1.115-1.151)	p < .0001
*Female*								
Continuous Q1	1 .023 (1 .014-1.033)	p < .0001	1.017 (1.006-1 .027)	p = .001	1.014 (1.004-1.024)	p = 005	1.021 (1.011-1.030)	p < .0001
Continuous Q2	1.093 (1.071-1.115)	p < 0001	1.048 (1.018-1.079)	p = .002	1.040 (1.013-1.069)	p = 004	1.079 (1.057-1.100)	p < .0001
Continuous Q3	1.096 (1.061-1.132)	p < .0001	1.074 (1.018-1.134)	p = .01	1.054 (1.009-1.100)	p = .02	1.081 (1.054-1.109)	p < .0001
Continuous Q4	1.167 (1.0918-1.240)	p < .0001	1.086 (0.999-1.181)	p = .05	1.050 (1.000-1.103)	p.05	1.061 (1.026-1.-097)	p < .0005

Adjusted for age group, marital status and occupation.

The overall finding is that the effect is larger in the reference groups defined by living region and by age group, and larger among men. Among men, there is no effect of relative deprivation on mortality within the lowest income quartile (Q1) regardless of the reference group used. However, there seems to be a threshold to the second lowest quartile (Q2) where the effect significantly differs from the lowest (Q1). Among women, only when defining reference group by living region there is a significant, although very small, effect.

## Discussion

Based on longitudinal data from the Swedish population with a mortality follow-up over a 16-year period, including a total of 4.7 million individuals aged 25–64 years, the present study makes a unique contribution to explaining the hypothesized mechanism of relative deprivation in the income-health relation. The primary findings of the present study are that relative income deprivation was significantly associated with premature mortality, irrespective of sociodemographic status, including individual income, and that this association was stronger among men than women. The effect of relative deprivation on mortality was weak among the poorest.

When analyzing relative deprivation as was done here, we do not know which reference groups are the most important. People tend to make a vast number of comparisons, but not all are important to feelings of relative deprivation and not all are important to health. In the present study, we used different types of objectively defined reference groups, all based on the idea that people compare themselves with similar others: same age group, same occupational group, same living region or a combination of these. Our results show an effect of relative deprivation on mortality for most of the tested reference groups, also after adjustments were made. These results resemble findings from previous studies showing relative deprivation to be important, although of different degree, irrespective of how reference groups were formed [5-8,13]. The Yitzhaki index is a measure of individual-level relative deprivation, albeit linked to income inequality as it is expected to increase with increasing disparities. Previous studies on the relation between income inequalities and health show mixed results, where the most consistent findings was reported in the United States when income distribution was measured at the state level [2]. However, analysing the importance of income inequality in Sweden, a previous study by Gerdtham and Johannesson did not find support for the relation to mortality [20]. Even though relative deprivation is linked to income inequality, the measure of relative deprivation captures individual level psychosocial impacts to a larger extent than for example the Gini index.

In line with previous results, we find that the effect of relative deprivation is more important for men than for women [6-8,13]. Eibner and Evans restricted their study to include only men, arguing that the measure of relative deprivation is not of equal importance to women due to their lower degree of labour market participation [15]. This could not be an argument in the Swedish context, although there may be other reasons why the measure lacks importance to women. In a study on pay reference standards and pay satisfaction, men were more affected by the national pay reference level and women more by the occupational level [21]. Comparing different measures of relative deprivation also showed that measures more closely related to everyday life and consumption affected women’s health more than measures of objective social status did. In a previous study a measure of self-rated deprivation, measuring a number of consumption items that the individual finds necessary but cannot afford, was found closely related to women’s health [8]. Among men, this is supported by the findings of Wolff and colleagues, showing that comparisons with distal reference groups impact health outcomes [22].

We also tried to isolate the effect of relative deprivation, over and above the effect of absolute income. The Yitzhaki index calculates the accumulated shortfall between one person’s income compared to all other with a higher income in his or her reference group. Considering this effect within different income strata give us an idea of the importance of social comparisons depending on income level. Among men, we did not find any significant effect of relative deprivation within the lowest income quartile (Table 3). A similar pattern was found in Japan by analysing relative deprivation and incident disability among the elderly [6], as well as in Sweden for the relation between relative deprivation and self-rated health [7]. It could be argued that psychosocial mechanisms, such as social comparisons, play a larger role when basic material needs are fulfilled. For individuals who are having problems making ends meet, relative deprivation may be of less importance.

The following limitations of the study should be considered. First, the way in which we formed our reference groups could be argued to be objective, thus not reflecting the groups people actually use for making social comparisons. Our data do not include any questions on people’s actual reference groups, rather we have based them on the assumption that people compare themselves to similar others – an argument that has also been used in other studies [5-8,13]. Based on previous studies, we would argue that our combinations of reference groups are sufficient for the present analyses.

Occupation was classified in accordance with the Swedish Socioeconomic Classification (SEI) and divided into the categories higher non-manual employees, lower non-manual employees, qualified manual workers, unqualified manual workers, self-employed and farmers. The total proportion of individuals who had an unclassifiable profession or had a missing value was 19 %, somewhat higher among women than men. These individuals were excluded from the analyses. This is likely to have impacted the results when using occupation as a reference group and could be one reason why the analysis of top vs. bottom within the lowest quartile showed a lower HR in the crude model when using occupation as a reference group. In the excluded group of people, those with an unclassifiable profession or a missing value, the proportion of individuals with a low income is likely to be higher. However, we performed analyses using different reference groups and all showed a similar pattern, namely that relative income deprivation is significantly associated with mortality.

There are reasons to believe that income inequality has an impact on individual health outcomes, and different mechanisms and explanations within this relation have been discussed [2,23]. Social comparisons’, generating relative deprivation, has been put forward as one possible mechanism. There are different hypotheses as to the breeding ground for relative deprivation. It has been argued that a context that promotes values of egalitarianism may in turn promote feelings of relative deprivation, as it encourages people to make comparisons with affluent others [11]. It could also be argued that psychosocial mechanisms may be more important in a setting where material deprivation, in an absolute sense, is not as common. However, even though these explanations seem important, this would not be as central for generating relative deprivation as large income disparities in a society.

## Conclusion

In sum, our study suggests that relative deprivation, based on social comparisons of income, is significantly associated with premature mortality in Sweden, over and above the effect of absolute income. Relative deprivation was found to be most important among men, although weak among the poorest individuals.

## Competing interests

The authors declare that they have no competing interests.

## Authors’ contribution

MÅY, NK, SH, IK all made substantial contributions to the manuscript. MÅY had the main responsibility for drafting the manuscript. NK and IK contributed substantially to the design of the study, revising the manuscript and interpretation of the results. SH performed the statistical analyses and contributed to the draft regarding methods and analyses. All authors read and approved the final version of the manuscript.

## Pre-publication history

The pre-publication history for this paper can be accessed here:

http://www.biomedcentral.com/1471-2458/12/664/prepub

## Supplementary Material

Additional file 1 Flow diagram over sample size. Click here for file
