# Supplementary material for: Relative deprivation and mortality – a longitudinal study in a Swedish population of 4,7 million, 1990–2006
Source: BMC Public Health. 2012 Aug 16;12:664. doi: 10.1186/1471-2458-12-664 (PMC3490850; doi:10.1186/1471-2458-12-664)
Supplement: Additional file 1 — Flow diagram over sample size. [file 1471-2458-12-664-S1.pptx]

## Slide 1
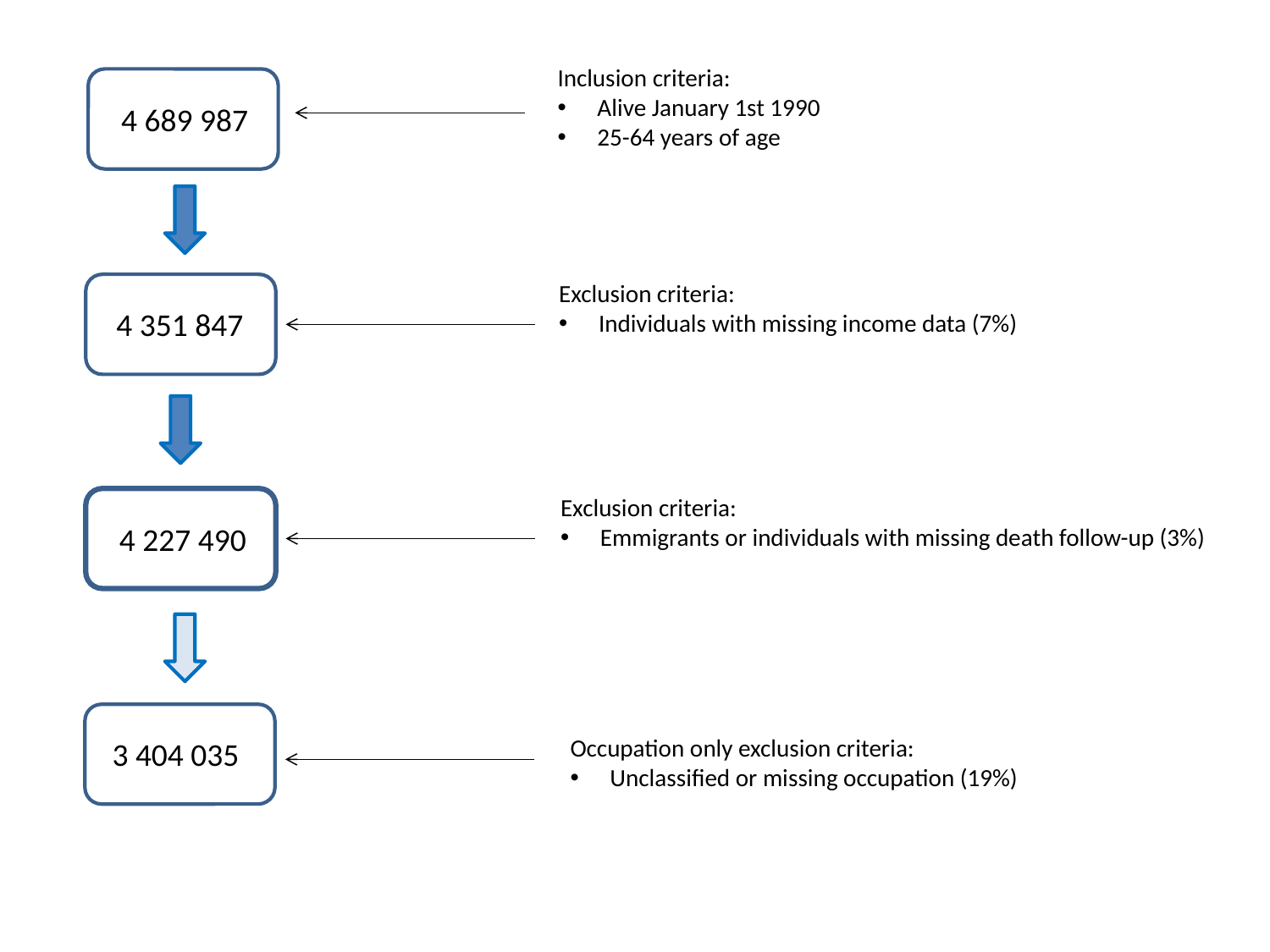

Inclusion criteria:
Alive January 1st 1990
25-64 years of age
4 689 987
Exclusion criteria:
Individuals with missing income data (7%)
4 351 847
Exclusion criteria:
Emmigrants or individuals with missing death follow-up (3%)
4 227 490
3 404 035
Occupation only exclusion criteria:
Unclassified or missing occupation (19%)
